# Genetic correlations and causal relationships between cardio-metabolic traits and sepsis

**DOI:** 10.1038/s41598-024-56467-7

**Published:** 2024-03-08

**Authors:** Zhongheng Zhang, Lin Chen, Haoyang Zhang, Wei Xiao, Jie Yang, Jiajie Huang, Qichao Hu, Ketao Jin, Yucai Hong

**Affiliations:** 1 Department of Emergency Medicine, Sir Run Run Shaw Hospital, Zhejiang University School of Medicine, Hangzhou, 310016 China; 2 Neurological Intensive Care Unit, Department of Neurosurgery, Affiliated Jinhua Hospital, Zhejiang University School of Medicine, Jinhua, China; 3 School of Computer Science and Engineering, Sun Yat-Sen University, Guangzhou, China; 4 Key Laboratory of Digital Technology in Medical Diagnostics of Zhejiang Province, Dian Diagnostics Group Co., Ltd., Hangzhou, Zhejiang China; 5 Department of Gastrointestinal, Colorectal and Anal Surgery, Affiliated Hangzhou First People’s Hospital, School of Medicine, Westlake University, Hangzhou, 310006 Zhejiang China

**Keywords:** Body mass index, Pathogenesis, Risk factors

## Abstract

Cardio-metabolic traits have been reported to be associated with the development of sepsis. It is, however, unclear whether these co-morbidities reflect causal associations, shared genetic heritability, or are confounded by environmental factors. We performed three analyses to explore the relationships between cardio-metabolic traits and sepsis. Mendelian randomization (MR) study to evaluate the causal effects of multiple cardio-metabolic traits on sepsis. Global genetic correlation analysis to explore the correlations between cardio-metabolic traits and sepsis. Local genetic correlation (GC) analysis to explore shared genetic heritability between cardio-metabolic traits and sepsis. Some loci were further examined for related genes responsible for the causal relationships. Genetic associations were obtained from the UK Biobank data or published large-scale genome-wide association studies with sample sizes between 200,000 to 750,000. In MR, we found causality between BMI and sepsis (OR: 1.53 [1.4–1.67]; p < 0.001). Body mass index (BMI), which is confirmed by sensitivity analyses and multivariable MR adjusting for confounding factors. Global GC analysis showed a significant correlation between BMI and sepsis (r_g_ = 0.55, p < 0.001). More cardio-metabolic traits were identified to be correlated to the sepsis onset such as CRP (rg = 0.37, p = 0.035), type 2 diabetes (r_g_ = 0.33, p < 0.001), HDL (r_g_ = − 0.41, p < 0.001), and coronary artery disease (r_g_ = 0.43, p < 0.001). Local GC revealed some shared genetic loci responsible for the causality. The top locus 1126 was located at chromosome 7 and comprised genes HIBADH, JAZF1, and CREB5. The present study provides evidence for an independent causal effect of BMI on sepsis. Further detailed analysis of the shared genetic heritability between cardio-metabolic traits and sepsis provides the opportunity to improve the preventive strategies for sepsis.

## Introduction

Sepsis is a leading cause of morbidity and mortality in hospitalized patients, with an estimated 48.9 million (95% uncertainty interval [UI] 38.9–62.9) incident cases and 11 million (10.1–12.0) sepsis-related deaths globally in 2017^1^. Although many efforts have been made to combat this syndrome, the clinical outcome remains suboptimal^2–4^. Since it is challenging to reduce the mortality rate for sepsis after the development of organ dysfunction, it would be interesting to initiate preventive measures for patients who are at risk of sepsis. Sepsis is caused by an uncontrolled inflammatory response to infection, and it is largely unknown why some patients are prone to sepsis while others are less likely to develop sepsis following infections. Thus, understanding risk factors for sepsis at the population level can be of vital importance to inform clinical decisions to prevent sepsis.

Since clinical cardio-metabolic traits are readily available for both hospitalized patients and the community population, understanding the linkages between some cardio-metabolic traits and sepsis predisposition would be interesting. There has been some evidence showing that certain cardio-metabolic traits such as obesity, type 2 diabetes, and C-reactive protein levels are associated with an increased risk of infectious complications^5–7^. However, most of these studies are performed in a special population such as those with inflammatory bowel disease, pregnancy, and major operations^8^. It remains unknown whether there are causal linkages between cardio-metabolic traits and sepsis at the population level. Furthermore, observational studies are prone to confounding bias and the causality between cardio-metabolic traits and sepsis is elusive^9^.

Individual genetic backgrounds such as genetic mutations (e.g., single nucleic polymorphism [SNP]) are responsible for sepsis predisposition and cardio-metabolic disorder. A genetic correlation is defined as the proportion of the heritability that is shared between two traits divided by the square root of the product of the heritability for each trait^10^. Based on the established genetic correlation, Mendelian randomization (MR) is a feasible choice for the investigation of causality. MR uses genetic variants to make a judgment about the causal nature of the relationship between a risk factor and an outcome based on observational data^11^. Although MR has been applied to explore the causality between some serum biomarkers and infectious diseases^12^, the shared genetics between cardio-metabolic traits and sepsis is still unclear.

In this study, we employed Mendelian randomization (MR) to explore the potential causal association between cardio-metabolic traits. Both local and global genetic correlations were also explored to confirm the association.

## Methods

### Study design and data sources

The study was conducted using summary-level statistics of the genome-wide association studies (GWAS) curated at the MRC Integrative Epidemiology Unit (IEU). To investigate the shared genetics between cardio-metabolic traits and sepsis in adult population, we selected the sepsis GWAS derived from the UK Biobank consortium and multiple cardio-metabolic traits derived from the CARDIoGRAMplusC4D and GIANT database^13–15^, to avoid sample overlap bias. Sepsis episodes were defined in the UK Biobank by hospital episode statistic primary or secondary International Classification of Diseases version 10 diagnosis codes as previously described (A021, A227, A327, A40, A41, A427, B377, O85, R651, and R572). If there is more than one GWAS for a given trait, we selected the GWAS with the largest sample sizes and consisting of the most similar populations. Sepsis was defined as the outcome trait. Other cardio-metabolic traits included body mass index (BMI), C-Reactive protein level, coronary artery disease, high-density lipoprotein (HDL) cholesterol, low-density lipoprotein (LDL) cholesterol levels, total cholesterol, type 2 diabetes, basophil cell count, diastolic blood pressure, systolic blood pressure, eosinophil cell count, lymphocyte cell count, monocyte cell count, neutrophil cell count, white blood cell count, and triglycerides (Table 1). Sensitivity analysis was performed by restricting to sepsis under 75 years old. Details on the study setting, participants selection, measurement, quality control and selection of genetic variants, and diagnostic criteria for traits can be found in original publications in Table 1.

**Table 1:** Data used for the Mendelian randomization analysis.

ID	Trait	Population	Sex	Category	Sample_size	nsnp	ncontrol	ncase	pmid
ieu-b-35	C-Reactive protein level	European	Males and females	Continuous	204,402	2,414,379	NA	NA	30,388,399
ebi-a-GCST005195	Coronary artery disease	NA	NA	NA	547,261	7,934,254	424,528	122,733	29,212,778
ieu-b-109	HDL cholesterol	European	Males and females	Continuous	403,943	12,321,875	NA	NA	32,203,549
ebi-a-GCST90002412	Low density lipoprotein cholesterol levels	European	NA	NA	431,167	16,293,344	NA	NA	32,493,714
ieu-b-4980	Sepsis	European	Males and females	Disease	486,484	12,243,539	474,841	11,643	NA
ieu-b-5066	Sepsis (under 75)	European	Males and females	Disease	462,869	12,243,540	451,301	11,568	NA
ieu-a-301	Total cholesterol	Mixed	Males and females	Risk factor	187,365	2,446,982	NA	NA	24,097,068
ebi-a-GCST006867	Type 2 diabetes	European	NA	NA	655,666	5,030,727	1178	61,714	30,054,458
ieu-b-29	Basophil cell count	European	Males and females	Continuous	563,946	NA	NA	NA	NA
ieu-b-40	Body mass index	European	Males and females	Continuous	681,275	2,336,260	NA	NA	30,124,842
ieu-b-39	Diastolic blood pressure	European	Males and females	Continuous	757,601	7,160,619	NA	NA	30,224,653
ieu-b-33	Eosinophil cell count	European	Males and females	Continuous	563,946	NA	NA	NA	NA
ieu-b-32	Lymphocyte cell count	European	Males and females	Continuous	563,946	NA	NA	NA	NA
ieu-b-31	Monocyte cell count	European	Males and females	Continuous	563,946	NA	NA	NA	NA
ieu-b-34	Neutrophil cell count	European	Males and females	Continuous	563,946	NA	NA	NA	NA
ieu-b-38	Systolic blood pressure	European	Males and females	Continuous	757,601	7,088,083	NA	NA	30,224,653
ieu-b-111	Triglycerides	European	Males and females	Continuous	441,016	12,321,875	NA	NA	32,203,549
ieu-b-30	White blood cell count	European	Males and females	Continuous	563,946	NA	NA	NA	NA

For categorical outcome data participant numbers were split into cases and controls.

### Causal inference with Mendelian randomization

Causal associations between cardio-metabolic traits and sepsis were first determined using the Mendelian randomization^11,16^. We assumed that there were no common causes (i.e. confounders) of the SNP(s) and sepsis, and there was no independent pathway between the SNP(s) and sepsis other than through the cardio-metabolic traits. Independent genetic variants (SNPs) strongly associated with cardio-metabolic traits were employed as the instrumental variables. To do so, we selected GWAS significant SNPs with p < 5 × 10^−8^ and then performed LD clumping with LD r^2^ < 0.001 within a 10,000 kb window. The secondary clumping threshold was *p* = 5 × 10^−8^. The extracted SNPs were then queried against the requested outcome of sepsis/sepsis (under 75). If a particular SNP is not present in the outcome dataset then it is possible to use SNPs that are LD ‘proxies’ instead. The proxies (LD tags) with minimum LD r2 value of 0.8 were looked for, and the tag alleles were aligned to target alleles. The effect of an SNP on an outcome and exposure were then harmonized to be relative to the same allele.

The heterogeneity statistics were reported to assess the robustness of the causal relationships. The result from each SNP was considered an independent RCT, and the results from all SNPs were pooled with a meta-analytic approach to obtain an overall causal estimate^17,18^. The effect size for each meta-analysis is reported in the main results as the effect of a one-standard deviation (1-SD) change in continuous traits (log transformation was applied if necessary). To examine whether the effect of BMI was independently associated with sepsis, we performed multivariable MR analysis. For each exposure, the instruments are selected then all exposures for those SNPs are regressed against the outcome together, weighting for the inverse variance of the outcome.

Pleiotropy is the phenomenon of a single genetic variant influencing multiple traits, which can lead to a false positive conclusion, we used multiple MR methods for the causal effect estimations, such as MR-Egger, weighted median, inverse variance weighted, simple mode, and weighted mode. We evaluated the directional pleiotropy based on the intercept obtained from the MR-Egger analysis^19^. We also performed a leave-one-out analysis in which we sequentially omitted one SNP at a time, to evaluate whether the MR estimate was driven or biased by a single SNP. The TwoSampleMR (v0.5.6) package was employed for this analysis. We follow the reporting guideline Strengthening the reporting of observational studies in epidemiology using the Mendelian randomization (STROBE-MR)^20^.

### Global genetic correlation analysis

The above-mentioned Mendelian randomization uses significantly associated SNPs as instrumental variables to quantify causal relationships between the exposure and outcome. This is effective for traits where many significant associations account for a substantial fraction of heritability. However, heritability is distributed over thousands of variants with small effects for many complex traits, thus genetic correlation was performed by using genome-wide data rather than data for only significantly associated variants to obtain more accurate results. Global genetic correlation (r_g_) analysis was performed using the cross-trait LD Score regression^10^. The method relies on the fact that the GWAS effect size estimate for a given SNP incorporates the effects of all SNPs in linkage disequilibrium (LD) with that SNP. For a polygenic trait, SNPs with high LD will have higher χ^2^ statistics on average than SNPs with low LD. A similar relationship holds if we replace the χ^2^ statistics for a single study with the product of the z scores from two studies of traits with non-zero genetic correlation. The python package LDSC (LD Score; v1.0.1) was employed for the analysis.

### Local genetic correlation analysis

A global r_g_ represents an average of the shared association across the genome, local r_g_s in opposing directions could result in a nonsignificant global r_g_, and local r_g_s in the absence of any global relation may be undetected. Thus, we performed local genetic correlation analysis by using the LAVA (Local Analysis of [co]Variant Association)^21^. Sample overlap was estimated using the intercepts from bivariate LDSC. The European panel of phase 3 of 1000 Genomes (MAF > 0.5%) was employed as an LD reference^22^. The genomic loci were created by partitioning the genome into blocks of approximately equal size (~ 1 Mb) while minimizing the LD between them. For each phenotype pair (traits versus sepsis), the loci were first filtered by the univariate test so that both phenotypes exhibited univariate signal at Holm-corrected P < 0.05. Multivariate genetic association analysis can be performed via either partial correlation or multiple regression. The analysis was performed by the R package LAVA (v0.1.0)^21^.

### Ethics approval and consent to participate

The study was conducted by secondary analysis of data from other studies, and informed consent was obtained from participants or their family members in the original studies.

## Results

### The causal association between cardio-metabolic traits and sepsis

**Figure 1:**
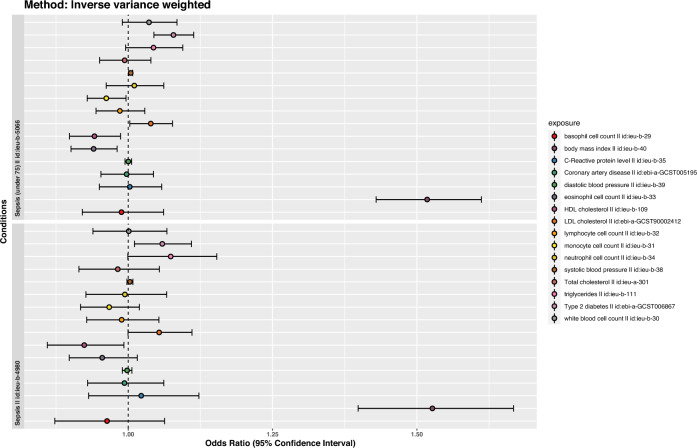
Forest plots showing the causal effects of cardio-metabolic traits on the risk of sepsis. inverse variance weights estimates were performed. Sensitivity analysis was performed by restricting to sepsis under 75 years old.

**Figure 2:**
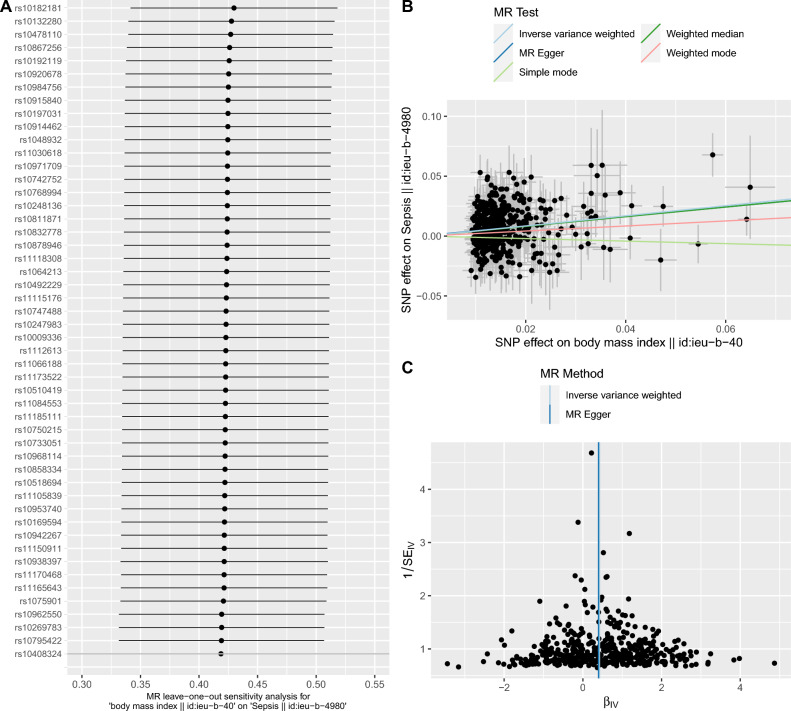
Sensitivity analyses to test the robustness of the results. (**A**) leave-one-off method to explore whether the effects can be driven by an individual SNP; (**B**) SNP effect on exposure and outcome. The slope of the line represents the causal effects of BMI on sepsis risk; (**C**) funnel plot showing the distribution of the effect of each SNP.

Genetically predicted larger BMI (each 1 SD increase) was associated with a significantly higher risk of sepsis (OR: 1.53 [1.4–1.67]; p < 0.001 by IVW method). As expected, the associations were consistent in sensitivity analyses using the MR-Egger method (OR: 1.49 [1.18–1.88]; p < 0.001) and the weighted median method (OR: 1.5 [1.29–1.74]; p < 0.001, Fig. 1). But the latter two methods provided less precise estimates than that with the conventional IVW method. In a leave-one-out sensitivity analysis, we found that no single SNP was strongly driving the overall effect of BMI on sepsis (Fig. 2A,C). The MR regression slopes are illustrated in Fig. 2B. There was no evidence for the presence of directional pleiotropy in the MR-Egger regression analysis, the P-values for the intercepts were large and the estimates adjusted for pleiotropy suggested null effects (Egger Intercept = 0.00047, p = 0.81; SDC Table S1). These results were in line with the hypothesis that genetic pleiotropy was not driving the result. No significant heterogeneity was identified for the causal effect of BMI on sepsis (Q = 511 for MR-Egger; p = 0.123; Q = 511 for IVW method, p = 0.129, SDC Table S2).

When we restricted to sepsis under 75 years old, the causal association between BMI and sepsis risk remains robust (OR: 1.52 [1.43–1.61]; p < 0.001; SDC Table S3 and Fig. 1). Consistently, the results were not driven by genetic pleiotropy (Egger Intercept = 0.00048, p = 0.72; SDC Table S2) and there was no significant heterogeneity (Q = 992.6 for MR-Egger; p = 0.175; Q = 992.8 for IVW method, p = 0.180).

Other cardio-metabolic traits that were associated with sepsis risk included type 2 diabetes (OR: 1.06 [1.01–1.11], p = 0.016 with IVW method), HDL (OR: 0.92 [0.86–0.99]; p = 0.031) and LDL (OR: 1.04 [1–1.08]; p = 0.035) cholesterol levels. Although these effects did not reach statistical significance using the Egger’s method (SDC Table S2), their causal estimates were similar in direction and magnitude, and they were unlikely to occur by chance alone.

**Figure 3:**
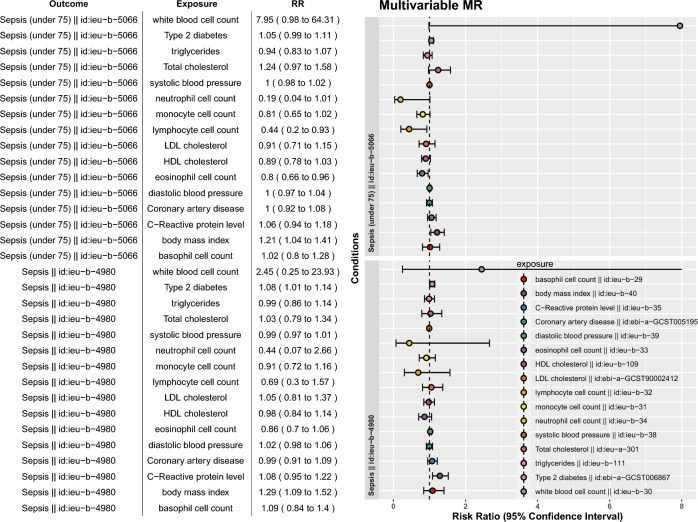
Multivariable MR analysis to adjust for possible confounding factors. The error bar indicates a 95% confidence interval.

To examine whether the effect of BMI was independently associated with sepsis, we performed multivariable MR analysis. The results showed that BMI was independently associated with sepsis risk (adjusted OR: 1.29; 95% CI: 1.09–1.52), while other cardio-metabolic traits were no longer associated with the sepsis risk (Fig. 3). Similar results were reproduced by restricting to sepsis under 75 years old (adjusted OR: 1.21; 95% CI: 1.04–1.41), although the magnitude was lower. This result indicated that the causal effects of type 2 diabetes, LDL, and HDL could be explained by BMI.

### Global genetic correlation analysis

**Figure 4:**
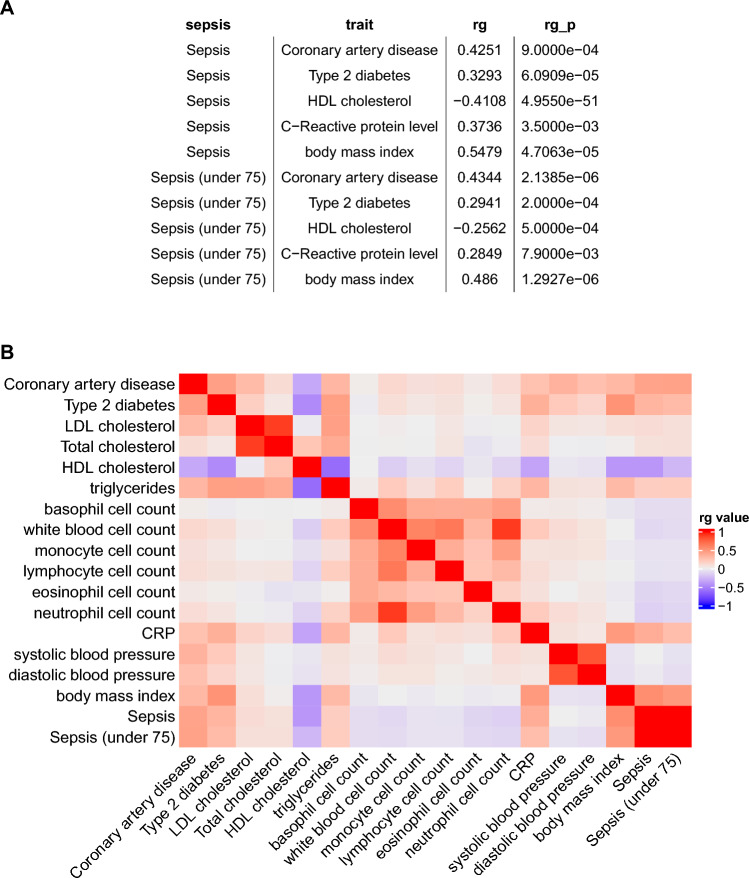
Global genetic correlations across sepsis and cardio-metabolic traits. (**A**) genetic correlation for top pairs of cardio-metabolic traits and sepsis; (**B**) Heatmap plot showing the genetic correlation across each pair of traits.

Since sepsis is a complex trait and its development is driven by thousands of genetic variants, with small effects from each of these variants. Thus, the genetic correlation was performed by using genome-wide data rather than data for only significantly associated variants to obtain more accurate results (SDC Table S4). As compared with the MR analysis, more cardio-metabolic traits were identified to be correlated to the sepsis onset such as CRP (r_g_ = 0.37, p = 0.035), type 2 diabetes (r_g_ = 0.33, p < 0.001), HDL (r_g_ = − 0.41, p < 0.001), coronary artery disease (r_g_ = 0.43, p < 0.001), and BMI (r_g_ = 0.55, p < 0.001). The results were consistent in sepsis under 75 (Fig. 4A). There were other cross-trait correlation pairs such as type 2 diabetes and HDL cholesterol, CRP and BMI (Fig. 4B).

### Local genetic correlation analysis

**Figure 5:**
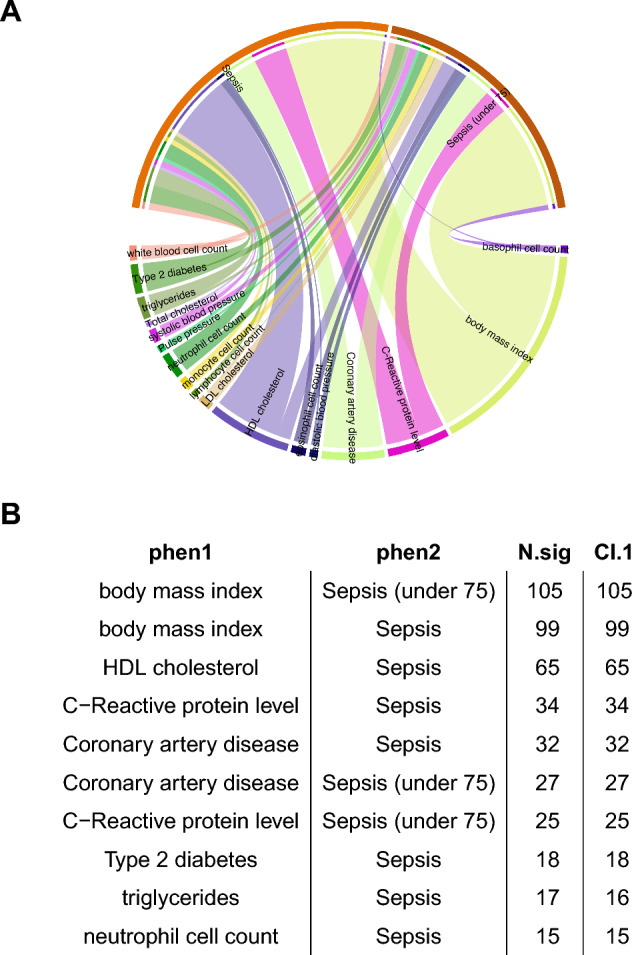
Local genetic correlation between sepsis and cardio-metabolic traits estimated by the LAVA method. (**A**) chord plot showing the correlation strength between cardio-metabolic traits and sepsis. the thickness of the line indicates the number of significant loci; (**B**) The number of significant loci for selected pairs of sepsis and cardio-metabolic traits. *CI.1* confidence interval includes 1, *N.sig* number of significant loci.

We applied LAVA to sepsis outcome and cardio-metabolic traits (Table 1), testing the pairwise local r_g_s within 2495 genomic loci (genome-wide). The genomic loci were created by partitioning the genome into blocks of approximately equal size (~ 1 Mb) while minimizing the LD between them, and the genomic coordinates are in reference to the human genome build 37. Sample overlap was estimated using the intercepts from bivariate LDSC obtained in the above section. With a Holm-corrected p < 0.05, we detected 572 significant bivariate local r_g_s across 318 loci, of which 140 loci were associated with more than one phenotype pair. Figure 5A shows the correlation between cardio-metabolic traits and sepsis outcome. The correlation strength as measured by the number of significant local r_g_s was consistent for sepsis and sepsis under 75. BMI showed the largest number of significant r_g_s, followed by HDL, CRP, and CAD. For most significant correlations, 95% confidence intervals (CIs) for the explained variance included 1, consistent with the scenario that the local genetic signal of those phenotypes is completely shared (Fig. 5B).

**Figure 6:**
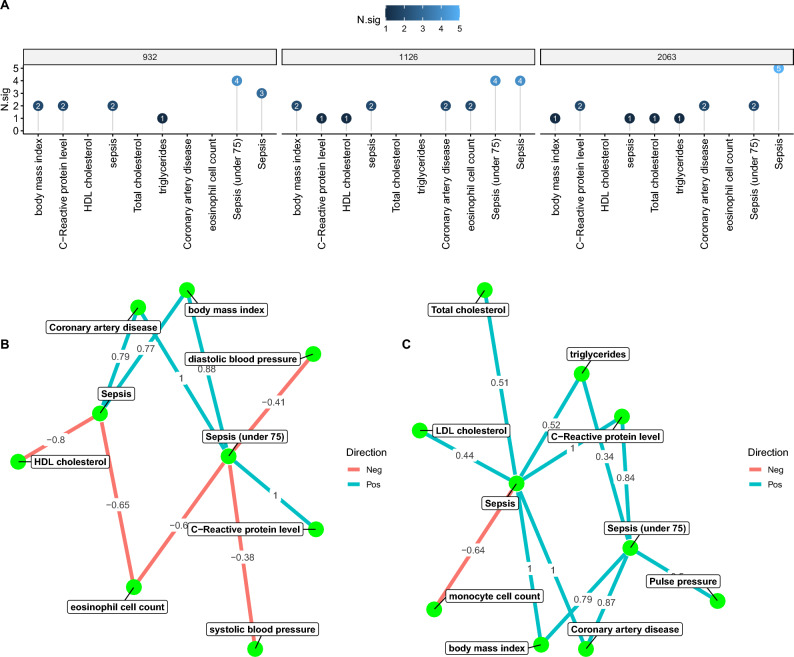
Sample loci with the top number of significant traits. (**A**) The top 3 loci with the largest number of significant traits; genetic correlation network between traits for locus 1126 (**B**) and 2036 (**C**). The red color indicates a negative correlation, and the blue color indicates a positive correlation. The number on the line indicates the genetic correlation (r_g_). Each green node represents a trait.

We further displayed three top loci that had the largest number of significant correlations to examine possible genes driving these traits (Fig. 6A–C). The locus 1126 had the greatest number of significant r_g_s, which showed positive r_g_s for BMI and CAD, and negative r_g_s for HDL and eosinophil cell count (Fig. 6B). The locus 1126 was located at chromosome 7 and comprised genes HIBADH (3-Hydroxyisobutyrate Dehydrogenase), JAZF1 (JAZF Zinc Finger 1), and CREB5 (CAMP Responsive Element Binding Protein 5). In particular, CREB5 is involved in the PI3K-Akt signaling pathway and Toll-like receptor signaling pathway, which has been widely explored in the sepsis^23,24^.

## Discussion

Our study found a causal effect of BMI on sepsis risk with MR analysis, and there was no evidence for the violation of IV assumptions with sophisticated sensitivity analyses. There were also significant genetic correlations between BMI and sepsis in both local and global GC analyses. The local GC analysis also helps to find some important loci that may play important roles in the development of sepsis. Other cardio-metabolic traits were also identified to have causal effects on sepsis such as type 2 diabetes, HDL cholesterol, CRP, and coronary artery disease. However, these traits are not consistent in all analyses, and their causal effects remain to be elucidated. The strengths and implications of our study included the following aspects. First, the sample sizes of each study are large, ranging from 200,000 to 750,000. The large sample sizes covered the representative population with sufficient statistical power for the GWAS. Second, the causal influence was estimated using the MR method using the genetic variants as the instrumental variable. Theoretically, the genetic variants are less likely to be affected by environmental confounding factors and the causal inference is more reliable. Third, the MR results were confirmed by both global and local GC analyses. While the MR analysis utilized only significant SNPs, for complex traits such as sepsis, there can be thousands of SNPs with small effects responsible for the heritability, thus global GC can help to address this issue.

Cardio-metabolic traits have been explored in other epidemiological studies for their associations with the risk of sepsis development and/or sepsis severity. For example, in a large multi-center cohort study, lower BMI (< 20 kg/m^2^) was associated with reduced mortality in patients with bloodstream infection^25^. A compelling body of evidence from MR studies has significantly contributed to our understanding of the relationship between obesity and sepsis^26,27^. The pathogenetic pathways connecting BMI or obesity to sepsis risk are multifaceted. Chronic low-grade inflammation, altered immune responses, and metabolic dysregulation have emerged as key contributors^28–30^. Studies have elucidated the impact of adipose tissue-derived inflammatory mediators on immune function, potentially predisposing obese individuals to an exaggerated inflammatory response during infections^31,32^. However, studies conducted in critical care settings showed that greater BMI was associated with improved survival, which is known as the obesity paradox in the intensive care unit (ICU)^33–35^. Probably, the pathophysiology of critical illness is different from those in the non-critical care setting. Critically ill patients are more likely to benefit from a greater BMI and long-term exposure to low-grade metabolic inflammation. Possible pathological mechanisms underlying the obesity paradox included higher energy reserves, inflammatory preconditioning, anti-inflammatory immune profile, and endotoxin neutralization^36^. Furthermore, our study focused on the sepsis predisposition rather than the mortality risk after the development of sepsis. It should be emphasized that susceptibility to sepsis is not equivalent to sepsis severity. Epidemiological studies for sepsis predisposition are usually performed in the patient population who are not critically ill, and long-term exposure to metabolic inflammation increases the risk of sepsis^37,38^.

Although the MR technique employed genetic variants as the IV, which is less likely to be affected by environmental confounding factors. Violations to other IV criteria are still great threats to causal inference, such as the pleiotropic effects of genetic variants. To account for this bias, we first employed Egger’s method, which failed to identify statistically significant pleiotropic effects. The results were robust in sensitivity analysis restricting to sepsis under 75. Then, we performed multivariable MR analysis using covariates known to be associated with sepsis such as CRP, type 2 diabetes, and neutrophil counts. After covariate adjustment, BMI remains to be independently associated with sepsis. furthermore, we also performed a leave-one-off analysis to test whether there are SNPs that significantly drive the results. The results revealed that there was no single SNP strongly driving the overall effect of BMI on sepsis.

Although MR analysis consistently showed causal effects of BMI on sepsis predisposition, it was not able to reveal underlying mechanisms responsible for the association. Local GC analysis may help to reveal some potential pathways mediating the linkage. By examining genes residing within the top loci, we identified some potential pathways related to inflammatory responses. For example, in the top locus 1126, we found several genes that are playing key roles in inflammatory responses including HIBADH, JAZF1, and CREB5. JAZF1 encodes a nuclear protein with three C2H2-type zinc fingers and functions as a transcriptional repressor. Genetic variations in this gene are correlated with decreased body mass index (BMI) and waist circumference^39,40^. Further experimental studies confirmed its important role in adipocyte differentiation, obesity, insulin resistance, and inflammation^41,42^.

In conclusion, our MR study establishes the causal effects of increased BMI on sepsis development. While more work is needed to understand the pathophysiology explaining these associations, an underlying derangement in inflammation should be suspected.

### Supplementary Information

Supplementary Tables.

## Data Availability

Data are available from the Biobank as detailed in Table 1. The link to the database is: https://gwas.mrcieu.ac.uk/.
