# Supplementary material for: Genetic correlations and causal relationships between cardio-metabolic traits and sepsis
Source: Sci Rep. 2024 Mar 8;14:5718. doi: 10.1038/s41598-024-56467-7 (PMC10923865; doi:10.1038/s41598-024-56467-7)
Supplement: Supplementary file 1 — Supplementary Tables. [file 41598_2024_56467_MOESM1_ESM.pdf]

Supplemental digital contents for

Genetic correlations and causal relationships between cardio-metabolic traits and sepsis

Zhongheng Zhang#

Department of Emergency Medicine, Sir Run Run Shaw Hospital, Zhejiang University School of Medicine, Hangzhou, 310016, China. Email: zh_zhang1984@zju.edu.cn

Lin Chen#

Neurological Intensive Care Unit, Department of Neurosurgery, Affiliated Jinhua Hospital, Zhejiang University School of Medicine, Jinhua, China. Email: chlin1986@163.com

Haoyang Zhang

School of Computer Science and Engineering, Sun Yat-Sen University, Guangzhou, China. Email: zhanghaoyang0@hotmail.com

Wei Xiao

Department of Emergency Medicine, Sir Run Run Shaw Hospital, Zhejiang University School of Medicine, Hangzhou, 310016, China. Email: z2xiaowei@zju.edu.cn

Jie Yang

Department of Emergency Medicine, Sir Run Run Shaw Hospital, Zhejiang University School of Medicine, Hangzhou, 310016, China. Email: 22218206@zju.edu.cn

Jiajie Huang

Department of Emergency Medicine, Sir Run Run Shaw Hospital, Zhejiang University School of Medicine, Hangzhou, 310016, China. Email: 22218207@zju.edu.cn

Qichao Hu
Key Laboratory of Digital Technology in Medical Diagnostics of Zhejiang Province, Dian Diagnostics Group Co., Ltd. Hangzhou, Zhejiang Province, China. Email: zyyyhqc@zju.edu.cn
Ketao Jin
Department of Gastrointestinal, Colorectal and Anal Surgery, Affiliated Hangzhou First People's Hospital, School of Medicine, Westlake University, Hangzhou, Zhejiang 310006, PR China. Email: jinketao2001@zju.edu.cn
Yucai Hong
Department of Emergency Medicine, Sir Run Run Shaw Hospital, Zhejiang University School of Medicine, Hangzhou, 310016, China.
# ZZ and LC contributed equally to this work and should be considered as co-first author.
Correspondence to:
Zhongheng Zhang (MD);
Department of Emergency Medicine, Sir Run Run Shaw Hospital, Zhejiang University School of Medicine, Hangzhou, 310016, China. Email: zh_zhang1984@zju.edu.cn

Table of Contents

Table S1. Pleiotropic effects assessed by the Egger intercept 4

Table S2. Heterogeneity statistics for MR estimates 6

Table S3. MR results for traits and sepsis 10

Table S4. Results from LDSC analysis 16

Table S1. Pleiotropic effects assessed by the Egger intercept

outcome	exposure	egger_intercept	se	pval
Sepsis || id:ieu-b-4980	Coronary artery disease || id:ebi-a-GCST005195	-7.624907e-03	0.0049898332	0.131834390
Sepsis (under 75) || id:ieu-b-5066	Coronary artery disease || id:ebi-a-GCST005195	-1.502968e-03	0.0035211787	0.670296304
Sepsis || id:ieu-b-4980	Type 2 diabetes || id:ebi-a-GCST006867	3.396944e-03	0.0039442673	0.390964803
Sepsis (under 75) || id:ieu-b-5066	Type 2 diabetes || id:ebi-a-GCST006867	6.861293e-03	0.0026346257	0.009852028
Sepsis || id:ieu-b-4980	Low density lipoprotein cholesterol levels || id:ebi-a-GCST90002412	5.708032e-04	0.0014272844	0.689498147
Sepsis (under 75) || id:ieu-b-5066	Low density lipoprotein cholesterol levels || id:ebi-a-GCST90002412	1.370252e-03	0.0009729818	0.159579300
Sepsis || id:ieu-b-4980	Total cholesterol || id:ieu-a-301	-4.415564e-03	0.0032798242	0.182062323
Sepsis (under 75) || id:ieu-b-5066	Total cholesterol || id:ieu-a-301	-2.586982e-03	0.0020665018	0.212504829
Sepsis || id:ieu-b-4980	HDL cholesterol || id:ieu-b-109	-3.197818e-03	0.0016313927	0.050848066
Sepsis (under 75) || id:ieu-b-5066	HDL cholesterol || id:ieu-b-109	-2.970854e-03	0.0010653861	0.005452755
Sepsis || id:ieu-b-4980	triglycerides || id:ieu-b-111	3.007001e-03	0.0016475727	0.069069148
Sepsis (under 75) || id:ieu-b-5066	triglycerides || id:ieu-b-111	2.355312e-03	0.0010918139	0.031417297
Sepsis || id:ieu-b-4980	basophil cell count || id:ieu-b-29	-5.515146e-06	0.0023524431	0.998131927
Sepsis (under 75) || id:ieu-b-5066	basophil cell count || id:ieu-b-29	-9.561529e-05	0.0017018184	0.955225078
Sepsis || id:ieu-b-4980	white blood cell count || id:ieu-b-30	2.058647e-03	0.0015868131	0.195172155
Sepsis (under 75) || id:ieu-b-5066	white blood cell count || id:ieu-b-30	2.231359e-03	0.0011316586	0.048940240
Sepsis || id:ieu-b-4980	monocyte cell count || id:ieu-b-31	1.472980e-03	0.0013344256	0.270233784
Sepsis (under 75) || id:ieu-b-5066	monocyte cell count || id:ieu-b-31	1.049036e-03	0.0008833097	0.235284120
Sepsis || id:ieu-b-4980	lymphocyte cell count || id:ieu-b-32	1.064920e-03	0.0016121856	0.509227351

Sepsis (under 75) || id:ieu-b-5066	lymphocyte cell count || id:ieu-b-32	1.665002e-03	0.0010898727	0.126921174
Sepsis || id:ieu-b-4980	eosinophil cell count || id:ieu-b-33	2.909164e-03	0.0017120268	0.090045408
Sepsis (under 75) || id:ieu-b-5066	eosinophil cell count || id:ieu-b-33	2.404079e-03	0.0011793352	0.041825461
Sepsis || id:ieu-b-4980	neutrophil cell count || id:ieu-b-34	1.061408e-04	0.0017200903	0.950827984
Sepsis (under 75) || id:ieu-b-5066	neutrophil cell count || id:ieu-b-34	-9.033837e-04	0.0012056778	0.453915889
Sepsis || id:ieu-b-4980	C-Reactive protein level || id:ieu-b-35	2.491974e-03	0.0040258867	0.538679686
Sepsis (under 75) || id:ieu-b-5066	C-Reactive protein level || id:ieu-b-35	-9.359418e-04	0.0023158482	0.686934924
Sepsis || id:ieu-b-4980	systolic blood pressure || id:ieu-b-38	-8.854039e-04	0.0020362944	0.663922161
Sepsis (under 75) || id:ieu-b-5066	systolic blood pressure || id:ieu-b-38	-6.660858e-04	0.0013430421	0.620056686
Sepsis || id:ieu-b-4980	diastolic blood pressure || id:ieu-b-39	-8.659759e-04	0.0018741405	0.644267973
Sepsis (under 75) || id:ieu-b-5066	diastolic blood pressure || id:ieu-b-39	4.632451e-04	0.0012841181	0.718375005
Sepsis || id:ieu-b-4980	body mass index || id:ieu-b-40	4.674823e-04	0.0019272542	0.808447963
Sepsis (under 75) || id:ieu-b-5066	body mass index || id:ieu-b-40	4.798772e-04	0.0013140627	0.715053763

Table S2. Heterogeneity statistics for MR estimates

outcome	exposure	method	Q	Q_df	Q_pval
Sepsis (under 75) || id:ieu-b-5066	Coronary artery disease || id:ebi-a-GCST005195	Inverse variance weighted	151.72757	116	0.014506234
Sepsis (under 75) || id:ieu-b-5066	Type 2 diabetes || id:ebi-a-GCST006867	Inverse variance weighted	280.14378	215	0.001853379
Sepsis (under 75) || id:ieu-b-5066	Low density lipoprotein cholesterol levels || id:ebi-a-GCST90002412	Inverse variance weighted	668.60749	579	0.005738515
Sepsis (under 75) || id:ieu-b-5066	Total cholesterol || id:ieu-a-301	Inverse variance weighted	141.40085	156	0.792677352
Sepsis (under 75) || id:ieu-b-5066	HDL cholesterol || id:ieu-b-109	Inverse variance weighted	696.65017	639	0.056477407
Sepsis (under 75) || id:ieu-b-5066	triglycerides || id:ieu-b-111	Inverse variance weighted	581.23934	553	0.196258591
Sepsis (under 75) || id:ieu-b-5066	basophil cell count || id:ieu-b-29	Inverse variance weighted	408.07170	375	0.115458256
Sepsis (under 75) || id:ieu-b-5066	white blood cell count || id:ieu-b-30	Inverse variance weighted	971.17125	909	0.074694839
Sepsis (under 75) || id:ieu-b-5066	monocyte cell count || id:ieu-b-31	Inverse variance weighted	910.74854	939	0.739936639
Sepsis (under 75) || id:ieu-b-5066	lymphocyte cell count || id:ieu-b-32	Inverse variance weighted	893.90226	945	0.881363967
Sepsis (under 75) || id:ieu-b-5066	eosinophil cell count || id:ieu-b-33	Inverse variance weighted	870.84367	807	0.058724761
Sepsis (under 75) || id:ieu-b-5066	neutrophil cell count || id:ieu-b-34	Inverse variance weighted	859.26162	787	0.037119223
Sepsis (under 75) || id:ieu-b-5066	C-Reactive protein level || id:ieu-b-35	Inverse variance weighted	121.98218	105	0.123125765
Sepsis (under 75) || id:ieu-b-5066	systolic blood pressure || id:ieu-b-38	Inverse variance weighted	829.56486	845	0.641148837

Sepsis (under 75) || id:ieu-b-5066	diastolic blood pressure || id:ieu-b-39	Inverse variance weighted	806.20195	863	0.916545180
Sepsis (under 75) || id:ieu-b-5066	body mass index || id:ieu-b-40	Inverse variance weighted	992.77033	953	0.180410136
Sepsis (under 75) || id:ieu-b-5066	Coronary artery disease || id:ebi-a-GCST005195	MR Egger	151.48757	115	0.012771529
Sepsis (under 75) || id:ieu-b-5066	Type 2 diabetes || id:ebi-a-GCST006867	MR Egger	271.53798	214	0.004708623
Sepsis (under 75) || id:ieu-b-5066	Low density lipoprotein cholesterol levels || id:ebi-a-GCST90002412	MR Egger	666.32112	578	0.006290604
Sepsis (under 75) || id:ieu-b-5066	Total cholesterol || id:ieu-a-301	MR Egger	139.83368	155	0.803173491
Sepsis (under 75) || id:ieu-b-5066	HDL cholesterol || id:ieu-b-109	MR Egger	688.26173	638	0.082224362
Sepsis (under 75) || id:ieu-b-5066	triglycerides || id:ieu-b-111	MR Egger	576.38009	552	0.228675733
Sepsis (under 75) || id:ieu-b-5066	basophil cell count || id:ieu-b-29	MR Egger	408.06826	374	0.108615318
Sepsis (under 75) || id:ieu-b-5066	white blood cell count || id:ieu-b-30	MR Egger	967.03066	908	0.085054184
Sepsis (under 75) || id:ieu-b-5066	monocyte cell count || id:ieu-b-31	MR Egger	909.33810	938	0.743177457
Sepsis (under 75) || id:ieu-b-5066	lymphocyte cell count || id:ieu-b-32	MR Egger	891.56838	944	0.887694457
Sepsis (under 75) || id:ieu-b-5066	eosinophil cell count || id:ieu-b-33	MR Egger	866.37689	806	0.068863584
Sepsis (under 75) || id:ieu-b-5066	neutrophil cell count || id:ieu-b-34	MR Egger	858.64832	786	0.036294222
Sepsis (under 75) || id:ieu-b-5066	C-Reactive protein level || id:ieu-b-35	MR Egger	121.79090	104	0.112156942
Sepsis (under 75) || id:ieu-b-5066	systolic blood pressure || id:ieu-b-38	MR Egger	829.31889	844	0.634266510

Sepsis (under 75) || id:ieu-b-5066	diastolic blood pressure || id:ieu-b-39	MR Egger	806.07181	862	0.913262741
Sepsis (under 75) || id:ieu-b-5066	body mass index || id:ieu-b-40	MR Egger	992.63127	952	0.175290185
Sepsis || id:ieu-b-4980	Coronary artery disease || id:ebi-a-GCST005195	Inverse variance weighted	81.01250	60	0.036688592
Sepsis || id:ieu-b-4980	Type 2 diabetes || id:ebi-a-GCST006867	Inverse variance weighted	155.79688	112	0.003943788
Sepsis || id:ieu-b-4980	Low density lipoprotein cholesterol levels || id:ebi-a-GCST90002412	Inverse variance weighted	370.83543	301	0.003697171
Sepsis || id:ieu-b-4980	Total cholesterol || id:ieu-a-301	Inverse variance weighted	100.26666	80	0.062310558
Sepsis || id:ieu-b-4980	HDL cholesterol || id:ieu-b-109	Inverse variance weighted	390.69071	319	0.003728371
Sepsis || id:ieu-b-4980	triglycerides || id:ieu-b-111	Inverse variance weighted	317.54052	276	0.043220827
Sepsis || id:ieu-b-4980	basophil cell count || id:ieu-b-29	Inverse variance weighted	182.31801	187	0.583008868
Sepsis || id:ieu-b-4980	white blood cell count || id:ieu-b-30	Inverse variance weighted	456.35907	454	0.460064975
Sepsis || id:ieu-b-4980	monocyte cell count || id:ieu-b-31	Inverse variance weighted	513.16670	469	0.077692702
Sepsis || id:ieu-b-4980	lymphocyte cell count || id:ieu-b-32	Inverse variance weighted	494.69819	472	0.226971679
Sepsis || id:ieu-b-4980	eosinophil cell count || id:ieu-b-33	Inverse variance weighted	439.90576	403	0.099296194
Sepsis || id:ieu-b-4980	neutrophil cell count || id:ieu-b-34	Inverse variance weighted	417.72110	393	0.187379738
Sepsis || id:ieu-b-4980	C-Reactive protein level || id:ieu-b-35	Inverse variance weighted	87.24648	52	0.001590383
Sepsis || id:ieu-b-4980	systolic blood pressure || id:ieu-b-38	Inverse variance weighted	464.31055	422	0.075908577

Sepsis || id:ieu-b-4980	diastolic blood pressure || id:ieu-b-39	Inverse variance weighted	439.47552	431	0.378478618
Sepsis || id:ieu-b-4980	body mass index || id:ieu-b-40	Inverse variance weighted	511.01674	476	0.129304360
Sepsis || id:ieu-b-4980	Coronary artery disease || id:ebi-a-GCST005195	MR Egger	77.92832	59	0.050017442
Sepsis || id:ieu-b-4980	Type 2 diabetes || id:ebi-a-GCST006867	MR Egger	154.76272	111	0.003853988
Sepsis || id:ieu-b-4980	Low density lipoprotein cholesterol levels || id:ebi-a-GCST90002412	MR Egger	370.63783	300	0.003354764
Sepsis || id:ieu-b-4980	Total cholesterol || id:ieu-a-301	MR Egger	98.01787	79	0.072380954
Sepsis || id:ieu-b-4980	HDL cholesterol || id:ieu-b-109	MR Egger	386.02648	318	0.005355240
Sepsis || id:ieu-b-4980	triglycerides || id:ieu-b-111	MR Egger	313.74024	275	0.053891042
Sepsis || id:ieu-b-4980	basophil cell count || id:ieu-b-29	MR Egger	182.31800	186	0.562592700
Sepsis || id:ieu-b-4980	white blood cell count || id:ieu-b-30	MR Egger	454.66975	453	0.469093062
Sepsis || id:ieu-b-4980	monocyte cell count || id:ieu-b-31	MR Egger	511.83413	468	0.078981709
Sepsis || id:ieu-b-4980	lymphocyte cell count || id:ieu-b-32	MR Egger	494.24034	471	0.221618352
Sepsis || id:ieu-b-4980	eosinophil cell count || id:ieu-b-33	MR Egger	436.76857	402	0.112016409
Sepsis || id:ieu-b-4980	neutrophil cell count || id:ieu-b-34	MR Egger	417.71704	392	0.178055556
Sepsis || id:ieu-b-4980	C-Reactive protein level || id:ieu-b-35	MR Egger	86.59591	51	0.001373894
Sepsis || id:ieu-b-4980	systolic blood pressure || id:ieu-b-38	MR Egger	464.10214	421	0.072100970
Sepsis || id:ieu-b-4980	diastolic blood pressure || id:ieu-b-39	MR Egger	439.25742	430	0.368385172
Sepsis || id:ieu-b-4980	body mass index || id:ieu-b-40	MR Egger	510.95345	475	0.123067904

Table S3. MR results for traits and sepsis

outcome	exposure	method	OR_95CI	pval
Sepsis || id:ieu-b-4980	Coronary artery disease || id:ebi-a-GCST005195	MR Egger	1.08 [0.95-1.23]	0.227
Sepsis || id:ieu-b-4980	Coronary artery disease || id:ebi-a-GCST005195	Weighted median	0.97 [0.89-1.07]	0.583
Sepsis || id:ieu-b-4980	Coronary artery disease || id:ebi-a-GCST005195	Inverse variance weighted	0.99 [0.93-1.06]	0.848
Sepsis (under 75) || id:ieu-b-5066	Coronary artery disease || id:ebi-a-GCST005195	MR Egger	1.01 [0.93-1.11]	0.758
Sepsis (under 75) || id:ieu-b-5066	Coronary artery disease || id:ebi-a-GCST005195	Weighted median	0.95 [0.89-1.02]	0.187
Sepsis (under 75) || id:ieu-b-5066	Coronary artery disease || id:ebi-a-GCST005195	Inverse variance weighted	1 [0.95-1.04]	0.908
Sepsis || id:ieu-b-4980	Type 2 diabetes || id:ebi-a-GCST006867	MR Egger	1.02 [0.91-1.13]	0.771
Sepsis || id:ieu-b-4980	Type 2 diabetes || id:ebi-a-GCST006867	Weighted median	1.06 [1-1.13]	0.063
Sepsis || id:ieu-b-4980	Type 2 diabetes || id:ebi-a-GCST006867	Inverse variance weighted	1.06 [1.01-1.11]	0.016
Sepsis (under 75) || id:ieu-b-5066	Type 2 diabetes || id:ebi-a-GCST006867	MR Egger	0.99 [0.92-1.06]	0.821
Sepsis (under 75) || id:ieu-b-5066	Type 2 diabetes || id:ebi-a-GCST006867	Weighted median	1.05 [1-1.1]	0.051
Sepsis (under 75) || id:ieu-b-5066	Type 2 diabetes || id:ebi-a-GCST006867	Inverse variance weighted	1.08 [1.04-1.11]	<0.001
Sepsis || id:ieu-b-4980	Low density lipoprotein cholesterol levels || id:ebi-a-GCST90002412	MR Egger	1.04 [0.98-1.12]	0.211
Sepsis || id:ieu-b-4980	Low density lipoprotein cholesterol levels || id:ebi-a-GCST90002412	Weighted median	0.99 [0.92-1.07]	0.851

Sepsis || id:ieu-b-4980	Low density lipoprotein cholesterol levels || id:ebi-a-GCST90002412	Inverse variance weighted	1.05 [1-1.11]	0.051
Sepsis (under 75) || id:ieu-b-5066	Low density lipoprotein cholesterol levels || id:ebi-a-GCST90002412	MR Egger	1.02 [0.97-1.07]	0.448
Sepsis (under 75) || id:ieu-b-5066	Low density lipoprotein cholesterol levels || id:ebi-a-GCST90002412	Weighted median	0.99 [0.93-1.06]	0.849
Sepsis (under 75) || id:ieu-b-5066	Low density lipoprotein cholesterol levels || id:ebi-a-GCST90002412	Inverse variance weighted	1.04 [1-1.08]	0.035
Sepsis || id:ieu-b-4980	Total cholesterol || id:ieu-a-301	MR Egger	1.04 [0.93-1.17]	0.47
Sepsis || id:ieu-b-4980	Total cholesterol || id:ieu-a-301	Weighted median	0.97 [0.86-1.09]	0.593
Sepsis || id:ieu-b-4980	Total cholesterol || id:ieu-a-301	Inverse variance weighted	0.98 [0.91-1.05]	0.615
Sepsis (under 75) || id:ieu-b-5066	Total cholesterol || id:ieu-a-301	MR Egger	1.03 [0.96-1.1]	0.425
Sepsis (under 75) || id:ieu-b-5066	Total cholesterol || id:ieu-a-301	Weighted median	0.98 [0.91-1.06]	0.643
Sepsis (under 75) || id:ieu-b-5066	Total cholesterol || id:ieu-a-301	Inverse variance weighted	0.99 [0.95-1.04]	0.789
Sepsis || id:ieu-b-4980	HDL cholesterol || id:ieu-b-109	MR Egger	1.01 [0.9-1.12]	0.918
Sepsis || id:ieu-b-4980	HDL cholesterol || id:ieu-b-109	Weighted median	0.96 [0.86-1.08]	0.5
Sepsis || id:ieu-b-4980	HDL cholesterol || id:ieu-b-109	Inverse variance weighted	0.92 [0.86-0.99]	0.031
Sepsis (under 75) || id:ieu-b-5066	HDL cholesterol || id:ieu-b-109	MR Egger	1.02 [0.95-1.1]	0.612
Sepsis (under 75) || id:ieu-b-5066	HDL cholesterol || id:ieu-b-109	Weighted median	1.02 [0.94-1.1]	0.621
Sepsis (under 75) || id:ieu-b-5066	HDL cholesterol || id:ieu-b-109	Inverse variance weighted	0.94 [0.9-0.99]	0.012
Sepsis || id:ieu-b-4980	triglycerides || id:ieu-b-111	MR Egger	1 [0.89-1.11]	0.944

Sepsis || id:ieu-b-4980	triglycerides || id:ieu-b-111	Weighted median	1 [0.89-1.12]	0.946
Sepsis || id:ieu-b-4980	triglycerides || id:ieu-b-111	Inverse variance weighted	1.07 [1-1.15]	0.052
Sepsis (under 75) || id:ieu-b-5066	triglycerides || id:ieu-b-111	MR Egger	0.98 [0.92-1.06]	0.664
Sepsis (under 75) || id:ieu-b-5066	triglycerides || id:ieu-b-111	Weighted median	1 [0.92-1.09]	0.992
Sepsis (under 75) || id:ieu-b-5066	triglycerides || id:ieu-b-111	Inverse variance weighted	1.04 [1-1.09]	0.076
Sepsis || id:ieu-b-4980	basophil cell count || id:ieu-b-29	MR Egger	0.96 [0.8-1.17]	0.705
Sepsis || id:ieu-b-4980	basophil cell count || id:ieu-b-29	Weighted median	0.99 [0.84-1.17]	0.879
Sepsis || id:ieu-b-4980	basophil cell count || id:ieu-b-29	Inverse variance weighted	0.96 [0.87-1.06]	0.458
Sepsis (under 75) || id:ieu-b-5066	basophil cell count || id:ieu-b-29	MR Egger	0.99 [0.86-1.14]	0.907
Sepsis (under 75) || id:ieu-b-5066	basophil cell count || id:ieu-b-29	Weighted median	1.03 [0.92-1.16]	0.593
Sepsis (under 75) || id:ieu-b-5066	basophil cell count || id:ieu-b-29	Inverse variance weighted	0.99 [0.92-1.06]	0.748
Sepsis || id:ieu-b-4980	white blood cell count || id:ieu-b-30	MR Egger	0.93 [0.81-1.06]	0.262
Sepsis || id:ieu-b-4980	white blood cell count || id:ieu-b-30	Weighted median	0.96 [0.86-1.07]	0.473
Sepsis || id:ieu-b-4980	white blood cell count || id:ieu-b-30	Inverse variance weighted	1 [0.94-1.07]	0.975
Sepsis (under 75) || id:ieu-b-5066	white blood cell count || id:ieu-b-30	MR Egger	0.95 [0.87-1.05]	0.319
Sepsis (under 75) || id:ieu-b-5066	white blood cell count || id:ieu-b-30	Weighted median	1.01 [0.93-1.08]	0.888
Sepsis (under 75) || id:ieu-b-5066	white blood cell count || id:ieu-b-30	Inverse variance weighted	1.04 [0.99-1.08]	0.128

Sepsis || id:ieu-b-4980	monocyte cell count || id:ieu-b-31	MR Egger	0.93 [0.85-1.01]	0.104
Sepsis || id:ieu-b-4980	monocyte cell count || id:ieu-b-31	Weighted median	0.92 [0.85-1]	0.039
Sepsis || id:ieu-b-4980	monocyte cell count || id:ieu-b-31	Inverse variance weighted	0.97 [0.92-1.02]	0.214
Sepsis (under 75) || id:ieu-b-5066	monocyte cell count || id:ieu-b-31	MR Egger	0.94 [0.88-0.99]	0.024
Sepsis (under 75) || id:ieu-b-5066	monocyte cell count || id:ieu-b-31	Weighted median	0.95 [0.89-1.01]	0.096
Sepsis (under 75) || id:ieu-b-5066	monocyte cell count || id:ieu-b-31	Inverse variance weighted	0.96 [0.93-1]	0.03
Sepsis || id:ieu-b-4980	lymphocyte cell count || id:ieu-b-32	MR Egger	0.95 [0.83-1.09]	0.455
Sepsis || id:ieu-b-4980	lymphocyte cell count || id:ieu-b-32	Weighted median	0.9 [0.81-1.01]	0.064
Sepsis || id:ieu-b-4980	lymphocyte cell count || id:ieu-b-32	Inverse variance weighted	0.99 [0.93-1.05]	0.725
Sepsis (under 75) || id:ieu-b-5066	lymphocyte cell count || id:ieu-b-32	MR Egger	0.93 [0.85-1.01]	0.098
Sepsis (under 75) || id:ieu-b-5066	lymphocyte cell count || id:ieu-b-32	Weighted median	0.96 [0.89-1.03]	0.203
Sepsis (under 75) || id:ieu-b-5066	lymphocyte cell count || id:ieu-b-32	Inverse variance weighted	0.99 [0.94-1.03]	0.508
Sepsis || id:ieu-b-4980	eosinophil cell count || id:ieu-b-33	MR Egger	0.87 [0.78-0.98]	0.028
Sepsis || id:ieu-b-4980	eosinophil cell count || id:ieu-b-33	Weighted median	0.94 [0.85-1.03]	0.197
Sepsis || id:ieu-b-4980	eosinophil cell count || id:ieu-b-33	Inverse variance weighted	0.96 [0.9-1.02]	0.144
Sepsis (under 75) || id:ieu-b-5066	eosinophil cell count || id:ieu-b-33	MR Egger	0.87 [0.81-0.95]	0.001
Sepsis (under 75) || id:ieu-b-5066	eosinophil cell count || id:ieu-b-33	Weighted median	0.96 [0.9-1.02]	0.184

Sepsis (under 75) || id:ieu-b-5066	eosinophil cell count || id:ieu-b-33	Inverse variance weighted	0.94 [0.9-0.98]	0.004
Sepsis || id:ieu-b-4980	neutrophil cell count || id:ieu-b-34	MR Egger	0.99 [0.86-1.14]	0.896
Sepsis || id:ieu-b-4980	neutrophil cell count || id:ieu-b-34	Weighted median	0.96 [0.85-1.07]	0.456
Sepsis || id:ieu-b-4980	neutrophil cell count || id:ieu-b-34	Inverse variance weighted	0.99 [0.93-1.07]	0.874
Sepsis (under 75) || id:ieu-b-5066	neutrophil cell count || id:ieu-b-34	MR Egger	1.05 [0.94-1.16]	0.391
Sepsis (under 75) || id:ieu-b-5066	neutrophil cell count || id:ieu-b-34	Weighted median	1.04 [0.96-1.13]	0.34
Sepsis (under 75) || id:ieu-b-5066	neutrophil cell count || id:ieu-b-34	Inverse variance weighted	1.01 [0.96-1.06]	0.675
Sepsis || id:ieu-b-4980	C-Reactive protein level || id:ieu-b-35	MR Egger	0.99 [0.86-1.14]	0.893
Sepsis || id:ieu-b-4980	C-Reactive protein level || id:ieu-b-35	Weighted median	1.07 [0.95-1.2]	0.296
Sepsis || id:ieu-b-4980	C-Reactive protein level || id:ieu-b-35	Inverse variance weighted	1.02 [0.93-1.12]	0.639
Sepsis (under 75) || id:ieu-b-5066	C-Reactive protein level || id:ieu-b-35	MR Egger	1.01 [0.94-1.1]	0.717
Sepsis (under 75) || id:ieu-b-5066	C-Reactive protein level || id:ieu-b-35	Weighted median	1.02 [0.95-1.1]	0.568
Sepsis (under 75) || id:ieu-b-5066	C-Reactive protein level || id:ieu-b-35	Inverse variance weighted	1 [0.95-1.06]	0.92
Sepsis || id:ieu-b-4980	systolic blood pressure || id:ieu-b-38	MR Egger	1.01 [0.99-1.02]	0.395
Sepsis || id:ieu-b-4980	systolic blood pressure || id:ieu-b-38	Weighted median	1 [0.99-1.01]	0.678
Sepsis || id:ieu-b-4980	systolic blood pressure || id:ieu-b-38	Inverse variance weighted	1 [1-1.01]	0.258
Sepsis (under 75) || id:ieu-b-5066	systolic blood pressure || id:ieu-b-38	MR Egger	1.01 [1-1.02]	0.143

Sepsis (under 75) || id:ieu-b-5066	systolic blood pressure || id:ieu-b-38	Weighted median	1 [1-1.01]	0.19
Sepsis (under 75) || id:ieu-b-5066	systolic blood pressure || id:ieu-b-38	Inverse variance weighted	1 [1-1.01]	0.012
Sepsis || id:ieu-b-4980	diastolic blood pressure || id:ieu-b-39	MR Egger	1 [0.98-1.02]	0.813
Sepsis || id:ieu-b-4980	diastolic blood pressure || id:ieu-b-39	Weighted median	1 [0.99-1.01]	0.801
Sepsis || id:ieu-b-4980	diastolic blood pressure || id:ieu-b-39	Inverse variance weighted	1 [0.99-1.01]	0.657
Sepsis (under 75) || id:ieu-b-5066	diastolic blood pressure || id:ieu-b-39	MR Egger	1 [0.98-1.01]	0.766
Sepsis (under 75) || id:ieu-b-5066	diastolic blood pressure || id:ieu-b-39	Weighted median	1 [0.99-1.01]	0.919
Sepsis (under 75) || id:ieu-b-5066	diastolic blood pressure || id:ieu-b-39	Inverse variance weighted	1 [0.99-1.01]	0.941
Sepsis || id:ieu-b-4980	body mass index || id:ieu-b-40	MR Egger	1.49 [1.18-1.88]	<0.001
Sepsis || id:ieu-b-4980	body mass index || id:ieu-b-40	Weighted median	1.5 [1.29-1.74]	<0.001
Sepsis || id:ieu-b-4980	body mass index || id:ieu-b-40	Inverse variance weighted	1.53 [1.4-1.67]	<0.001
Sepsis (under 75) || id:ieu-b-5066	body mass index || id:ieu-b-40	MR Egger	1.48 [1.26-1.73]	<0.001
Sepsis (under 75) || id:ieu-b-5066	body mass index || id:ieu-b-40	Weighted median	1.4 [1.27-1.55]	<0.001
Sepsis (under 75) || id:ieu-b-5066	body mass index || id:ieu-b-40	Inverse variance weighted	1.52 [1.43-1.61]	<0.001

Table S4. Results from LDSC analysis

Outcome	Exposure	nsnp	h2_1	h2_1_se	Lambda_GC_1	MeanChi2_1	int_1	int_1_se	Ratio_1	Ratio_1_se	h2_2	h2_2_se	Lambda_GC_2	MeanChi2_2	int_2	int_2_se	Ratio_2	Ratio_2_se	rg	rg_se	rg_p	int_bi	int_bi_se	h2_1_p	h2_2_p
Sepsis	Coronary artery disease	1081769	0.1033	0.0073	1.3101	1.4397	1.0308	0.0118	0.07	0.0268	0.0024	0.0010	1.0375	1.0385	1.0153	0.0065	0.3963	0.17	0.4251	0.1277	9.0000e-04	0.0467	0.0048	1.849471e-45	1.639507e-02
Sepsis	Type 2 diabetes	929681	0.0460	0.0030	1.4602	1.6833	1.0512	0.0188	0.075	0.0275	0.0033	0.0011	1.0375	1.0399	1.0056	0.0081	0.1401	0.2043	0.3293	0.0821	6.0909e-05	0.0627	0.0073	4.578745e-53	2.699796e-03
Sepsis	Low density lipoprotein cholesterol levels	1080250	0.1807	0.0200	1.6259	2.8654	1.2809	0.1067	0.1506	0.0572	0.0025	0.0010	1.0345	1.0386	1.0144	0.0065	0.3744	0.1692	0.1093	0.0765	1.5310e-01	-0.0065	0.0066	1.640024e-19	1.241933e-02
Sepsis	Total cholesterol	954607	0.2154	0.0432	1.0135	1.228	0.8606	0.0583	< 0		0.0028	0.0010	1.0375	1.039	1.0117	0.0069	0.3013	0.1778	0.0815	0.0809	3.1330e-01	-0.0043	0.0049	6.160672e-07	5.110261e-03
Sepsis	HDL cholesterol	961132	0.1945	0.0158	1.9218	3.0129	1.3982	0.1402	0.1978	0.0697	0.2140	0.0066	2.7872	3.9316	1.0156	0.028	0.0053	0.0096	-0.4108	0.0273	4.9550e-51	-0.2873	0.0188	7.989778e-35	1.250041e-230
Sepsis	triglycerides	1087275	0.1685	0.0178	1.7648	2.7756	1.2055	0.0768	0.1157	0.0433	0.0025	0.0010	1.0375	1.0386	1.0149	0.0064	0.3857	0.1671	0.1902	0.1259	1.3060e-01	0.0236	0.0081	2.899537e-21	1.241933e-02
Sepsis	basophil cell count	1087106	0.0642	0.0067	1.3135	1.7114	1.0954	0.0291	0.1342	0.0409	0.0025	0.0010	1.0375	1.0386	1.0148	0.0064	0.3837	0.1666	-0.0817	0.1067	4.4380e-01	0.0105	0.006	9.510109e-22	1.241933e-02
Sepsis	white blood cell count	1087128	0.1843	0.0115	1.9383	3.3968	1.2787	0.0509	0.1163	0.0212	0.0025	0.0010	1.0375	1.0385	1.0148	0.0065	0.3849	0.1677	-0.103	0.0702	1.4250e-01	0.0275	0.007	8.400895e-58	1.241933e-02
Sepsis	monocyte cell count	1087103	0.2019	0.0197	1.7609	3.4676	1.4308	0.0906	0.1746	0.0367	0.0025	0.0010	1.0375	1.0385	1.0148	0.0064	0.3842	0.1669	-0.058	0.0665	3.8320e-01	0.012	0.0064	1.199074e-24	1.241933e-02
Sepsis	lymphocyte cell count	1087139	0.1991	0.0143	1.9013	3.4254	1.2739	0.0641	0.1129	0.0264	0.0025	0.0010	1.0375	1.0385	1.0148	0.0064	0.3837	0.167	-0.0576	0.0599	3.3670e-01	0.0008	0.0067	4.587228e-44	1.241933e-02
Sepsis	eosinophil cell count	1087079	0.1954	0.0203	1.6754	3.2399	1.2381	0.0638	0.1063	0.0285	0.0025	0.0010	1.0375	1.0385	1.0148	0.0064	0.3833	0.1666	-0.1171	0.0793	1.3990e-01	0.0108	0.0073	6.233246e-22	1.241933e-02
Sepsis	neutrophil cell count	1087134	0.1552	0.0111	1.7569	2.881	1.2194	0.0452	0.1167	0.024	0.0025	0.0010	1.0375	1.0385	1.0149	0.0065	0.386	0.1677	-0.1372	0.0787	8.1400e-02	0.0309	0.0071	2.008166e-44	1.241933e-02
Sepsis	C-Reactive protein level	978498	0.1139	0.0224	1.2531	1.5001	1.0296	0.046	0.0591	0.092	0.0027	0.0010	1.0375	1.0389	1.0131	0.007	0.3361	0.181	0.3736	0.1281	3.5000e-03	0.009	0.0071	3.679721e-07	6.933948e-03
Sepsis	systolic blood pressure	1079264	0.1403	0.0060	2.0812	3.1261	1.0852	0.032	0.0401	0.015	0.0026	0.0010	1.0375	1.0386	1.0137	0.0065	0.3537	0.1694	-0.0017	0.0738	9.8130e-01	0.0063	0.0072	6.315594e-121	9.322376e-03
Sepsis	diastolic blood pressure	1080848	0.1358	0.0062	2.0344	3.1317	1.1016	0.0329	0.0477	0.0154	0.0026	0.0010	1.0345	1.0386	1.0135	0.0066	0.3501	0.1706	-0.0265	0.0668	6.9120e-01	0.0089	0.0068	2.420249e-106	9.322376e-03
Sepsis	body mass index	961201	0.2140	0.0066	2.7872	3.9316	1.0156	0.0283	0.0053	0.0097	0.0023	0.0010	1.0375	1.0402	1.0177	0.0068	0.4409	0.1696	0.5479	0.1346	4.7063e-05	0.0283	0.0088	1.250041e-230	2.144822e-02
Sepsis (under 75)	Coronary artery disease	1081768	0.1033	0.0073	1.3101	1.4397	1.0308	0.0118	0.07	0.0268	0.0070	0.0021	1.0345	1.0332	1.0049	0.0058	0.1463	0.175	0.4344	0.0917	2.1385e-06	0.0398	0.005	1.849471e-45	8.581207e-04
Sepsis (under 75)	Type 2 diabetes	929681	0.0460	0.0030	1.4602	1.6833	1.0512	0.0188	0.075	0.0275	0.0079	0.0024	1.0375	1.0354	1.0008	0.0078	0.0213	0.2215	0.2941	0.08	2.0000e-04	0.0653	0.0067	4.578745e-53	9.959558e-04
Sepsis (under 75)	Low density lipoprotein cholesterol levels	1080250	0.1807	0.0200	1.6259	2.8654	1.2809	0.1067	0.1506	0.0572	0.0072	0.0021	1.0345	1.033	1.0042	0.006	0.1278	0.1829	0.0894	0.0577	1.2160e-01	-0.0041	0.006	1.640024e-19	6.067668e-04
Sepsis (under 75)	Total cholesterol	954606	0.2154	0.0433	1.0135	1.228	0.8606	0.0583	< 0		0.0076	0.0022	1.0345	1.0338	1.0032	0.0067	0.0957	0.199	0.0878	0.0719	2.2160e-01	-0.0019	0.0048	6.538391e-07	5.512213e-04
Sepsis (under 75)	HDL cholesterol	1087277	0.2015	0.0155	1.9218	3.0001	1.3634	0.1183	0.1817	0.0591	0.0071	0.0022	1.0345	1.0332	1.0045	0.006	0.1372	0.1808	-0.2562	0.0738	5.0000e-04	-0.0369	0.0065	1.223433e-38	1.249763e-03
Sepsis (under 75)	triglycerides	1087274	0.1685	0.0178	1.7648	2.7756	1.2055	0.0768	0.1157	0.0433	0.0071	0.0022	1.0345	1.0332	1.0045	0.006	0.135	0.1804	0.1934	0.0805	1.6200e-02	0.0264	0.0069	2.899537e-21	1.249763e-03

Sepsis (under 75)	basophil cell count	1087105	0.0642	0.0067	1.3135	1.7114	1.0954	0.0291	0.1342	0.0409	0.0073	0.0022	1.0345	1.0331	1.0039	0.006	0.1175	0.1809	-0.0822	0.0886	3.5330e-01	0.0155	0.0059	9.510109e-22	9.060549e-04
Sepsis (under 75)	white blood cell count	1087127	0.1843	0.0115	1.9383	3.3968	1.2787	0.0509	0.1163	0.0212	0.0072	0.0022	1.0345	1.0331	1.004	0.006	0.121	0.1816	-0.0875	0.0602	1.4630e-01	0.0269	0.0064	8.400895e-58	1.065152e-03
Sepsis (under 75)	monocyte cell count	1087102	0.2019	0.0197	1.7609	3.4676	1.4308	0.0906	0.1746	0.0367	0.0072	0.0022	1.0345	1.0331	1.004	0.006	0.1201	0.181	-0.0615	0.0578	2.8670e-01	0.0107	0.0058	1.199074e-24	1.065152e-03
Sepsis (under 75)	lymphocyte cell count	1087138	0.1991	0.0143	1.9013	3.4254	1.2739	0.0641	0.1129	0.0264	0.0072	0.0022	1.0345	1.0331	1.004	0.006	0.1206	0.1816	-0.0542	0.0561	3.3410e-01	-0.0004	0.0066	4.587228e-44	1.065152e-03
Sepsis (under 75)	eosinophil cell count	1087078	0.1954	0.0203	1.6754	3.2399	1.2381	0.0638	0.1063	0.0285	0.0073	0.0022	1.0345	1.0331	1.0039	0.006	0.1182	0.1807	-0.0999	0.0698	1.5230e-01	0.0143	0.0076	6.233246e-22	9.060549e-04
Sepsis (under 75)	neutrophil cell count	1087133	0.1552	0.0111	1.7569	2.881	1.2194	0.0452	0.1167	0.024	0.0072	0.0022	1.0345	1.0331	1.004	0.006	0.1214	0.1816	-0.1119	0.0652	8.5900e-02	0.0307	0.0064	2.008166e-44	1.065152e-03
Sepsis (under 75)	C-Reactive protein level	978497	0.1139	0.0224	1.2531	1.5001	1.0296	0.046	0.0591	0.092	0.0074	0.0022	1.0345	1.0335	1.004	0.0065	0.1181	0.1934	0.2849	0.1072	7.9000e-03	0.0098	0.006	3.679721e-07	7.692282e-04
Sepsis (under 75)	systolic blood pressure	1079264	0.1403	0.0060	2.0812	3.1261	1.0852	0.032	0.0401	0.015	0.0072	0.0022	1.0345	1.0332	1.0042	0.0061	0.1259	0.1827	-0.0573	0.0641	3.7130e-01	0.0207	0.007	6.315594e-121	1.065152e-03
Sepsis (under 75)	diastolic blood pressure	1080848	0.1358	0.0062	2.0344	3.1317	1.1016	0.0329	0.0477	0.0154	0.0073	0.0022	1.0345	1.0331	1.0035	0.006	0.1068	0.1826	-0.0717	0.0586	2.2160e-01	0.0162	0.0068	2.420249e-106	9.060549e-04
Sepsis (under 75)	body mass index	942879	0.2112	0.0066	2.7872	3.93	1.0213	0.0271	0.0073	0.0093	0.0065	0.0022	1.0345	1.0344	1.008	0.0065	0.2334	0.1896	0.486	0.1004	1.2927e-06	0.0346	0.0079	1.090416e-224	3.131300e-03

Note: trait1 = exposure, trait2=outcome, h2_1 = observed scale h2 for trait 1 (Heritability of trait 1), h2_1_se = standard error for h2_1, int_1 = single-trait LD Score regression intercept for trait 1, MeanChi2 = mean chi-square statistic, Lambda GC is median(chi^2)/0.4549;

Ratio is (intercept-1)/(mean(chi^2)-1), which measures the proportion of the inflation in the mean chi^2 that the LD Score regression intercept ascribes to causes other than polygenic heritability. The value of ratio should be close to zero, though in practice values of 10-20% are not uncommon, probably due to sample/reference LD Score mismatch or model misspecification (e.g., low LD variants have slightly higher h^2 per SNP).

rg = genetic correlation; int_bi = cross-trait LD Score regression intercept.
